# Simultaneous quantification of eight hemoglobin adducts of genotoxic substances by isotope-dilution UHPLC-MS/MS

**DOI:** 10.1007/s00216-022-04143-y

**Published:** 2022-06-02

**Authors:** Fabian Gauch, Klaus Abraham, Bernhard H. Monien

**Affiliations:** Department of Food Safety, German Federal Institute for Risk Assessment (BfR), Max-Dohrn-Str. 8-10, 10589 Berlin, Germany

**Keywords:** Hemoglobin adduct, Internal exposure, Edman degradation, Biomarker

## Abstract

Various genotoxic carcinogens ubiquitously present in the human environment or respective reactive metabolites form adducts in DNA and proteins, which can be used as biomarkers of internal exposure. For example, the mass spectrometric determination of Val adducts at the N-termini of hemoglobin (Hb) peptide chains after cleavage by an Edman degradation has a long tradition in occupational medicine. We developed a novel isotope-dilution UHPLC-MS/MS method for the simultaneous quantification of Val adducts of eight genotoxic substances in Hb after cleavage with fluorescein-5-isothiocyanate (FI*R*E procedure™). The following adducts were included [sources in square brackets]: *N*-(2,3-dihydroxypropyl)-Val [glycidol], *N*-(2-carbamoylethyl)-Val [acrylamide], *N*-(2-carbamoyl-2-hydroxyethyl)-Val [glycidamide], *N*-((furan-2-yl)methyl)-Val [furfuryl alcohol], *N*-(*trans*-isoestragole-3′-yl)-Val [estragole/anethole], *N*-(3-ketopentyl)-Val [1-penten-3-one], *N*-(3-ketooctanyl)-Val [1-octene-3-one], and *N*-benzyl-Val [benzyl chloride], each of which was quantified with a specific isotope-labeled standard. The limits of quantification were between 0.014 and 3.6 pmol/g Hb (using 35 mg Hb per analysis); other validation parameters were satisfactory according to guidelines of the U.S. Food and Drug Administration. The quantification in erythrocyte samples of human adults (proof of principle) showed that the median levels of Hb adducts of acrylamide, glycidamide, and glycidol were found to be significantly lower in six non-smokers (25.9, 12.2, and 4.7 pmol/g Hb, respectively) compared to those of six smokers (69.0, 44.2, and 8.6 pmol/g Hb, respectively). In summary, the method surpasses former techniques of Hb adduct quantification due to its simplicity, sensitivity, and accuracy. It can be extended continuously with other Hb adducts and will be used in epidemiological studies on internal exposure to carcinogens.

**Graphical abstract:**

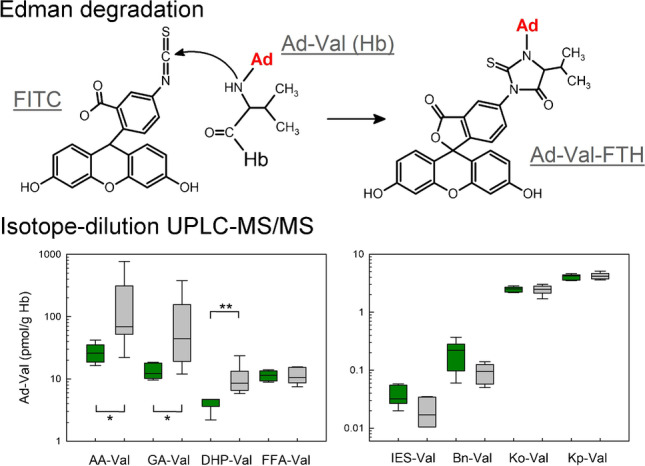

**Supplementary Information:**

The online version contains supplementary material available at 10.1007/s00216-022-04143-y.

## Introduction

Humans are exposed to genotoxic carcinogens in cigarette smoke and food. The carcinogens are natural constituents of plants (e.g., alkenylbenzenes in teas and herbs) [1], can be formed by heat (e.g., acrylamide and glycidol) [2], or occur as food contaminants, for example, as a result of fungal growth (mycotoxins) [3] or of environmental pollution (e.g., polycyclic aromatic hydrocarbons) [4]. The reactions of the substances with DNA (directly or after enzyme-catalyzed conversion into electrophilic metabolites) may induce the development of diseases, in particular cancer [5]. DNA adducts, however, are not suitable biomarkers for the internal exposure to reactive substances. The accessibility of human DNA is limited, and the lack of data on the lifetime of DNA adducts, most of which are removed by repair mechanisms, hinders deducing the carcinogen’s external exposure from a specific DNA adduct level [6]. The systemic exposure to the reactants is better monitored by protein adducts reflecting the internal exposure (or the biologically effective dose) to reactive molecules [7]. Common targets are human serum albumin (HSA) or hemoglobin (Hb), the latter of which was the preferred choice for protein adduct analysis in the last three decades. Hb is abundant (~ 150 mg/mL blood) and readily accessible and allows cumulative exposure monitoring due to the lifetime of approx. 120 days [8]. Assuming a continuous exposure in this timeframe leading to a steady-state level of a specific Hb adduct, it is possible to estimate this exposure from a single adduct quantification [9, 10].

The most common technique for the quantification of Hb adducts is the Edman degradation, the chemical cleavage of modified Val residues at the N-termini of α- and β-peptide chains of globin using isothiocyanates [11, 12]. A relatively recent method involves the application of fluorescein-5-isothiocyanate (FITC) in the so-called FI*R*E procedure™ leading to the formation of fluorescein thiohydantoin (FTH) derivatives (Fig. S1 of the Supplementary information), which are extracted with mixed-mode anion exchange columns and quantified by LC–MS/MS [12]. This renders the tedious globin isolation necessary for other Edman degradation methods obsolete, because the FITC–mediated Val cleavage is feasible using erythrocytes or even whole blood samples [13].

Lately, it was sought to extend the techniques devised for the quantification of single compound Hb adducts to allow the simultaneous quantification of multiple adducts. This is particularly reasonable considering that disease development is likely to be triggered by the internal exposure to a multitude of reactive compounds rather than single substances [14]. Schettgen et al. published a method for the analysis of five different Hb adducts by Edman degradation and isotope-dilution GC–MS [15]. Following the FITC–mediated cleavage, Carlsson et al. reported on a semi-quantitative method, in which levels of 12 acknowledged adducts were estimated using one deuterated standard compound. Further, 12 additional adducts yet unknown were described [16].

**Fig. 1:**
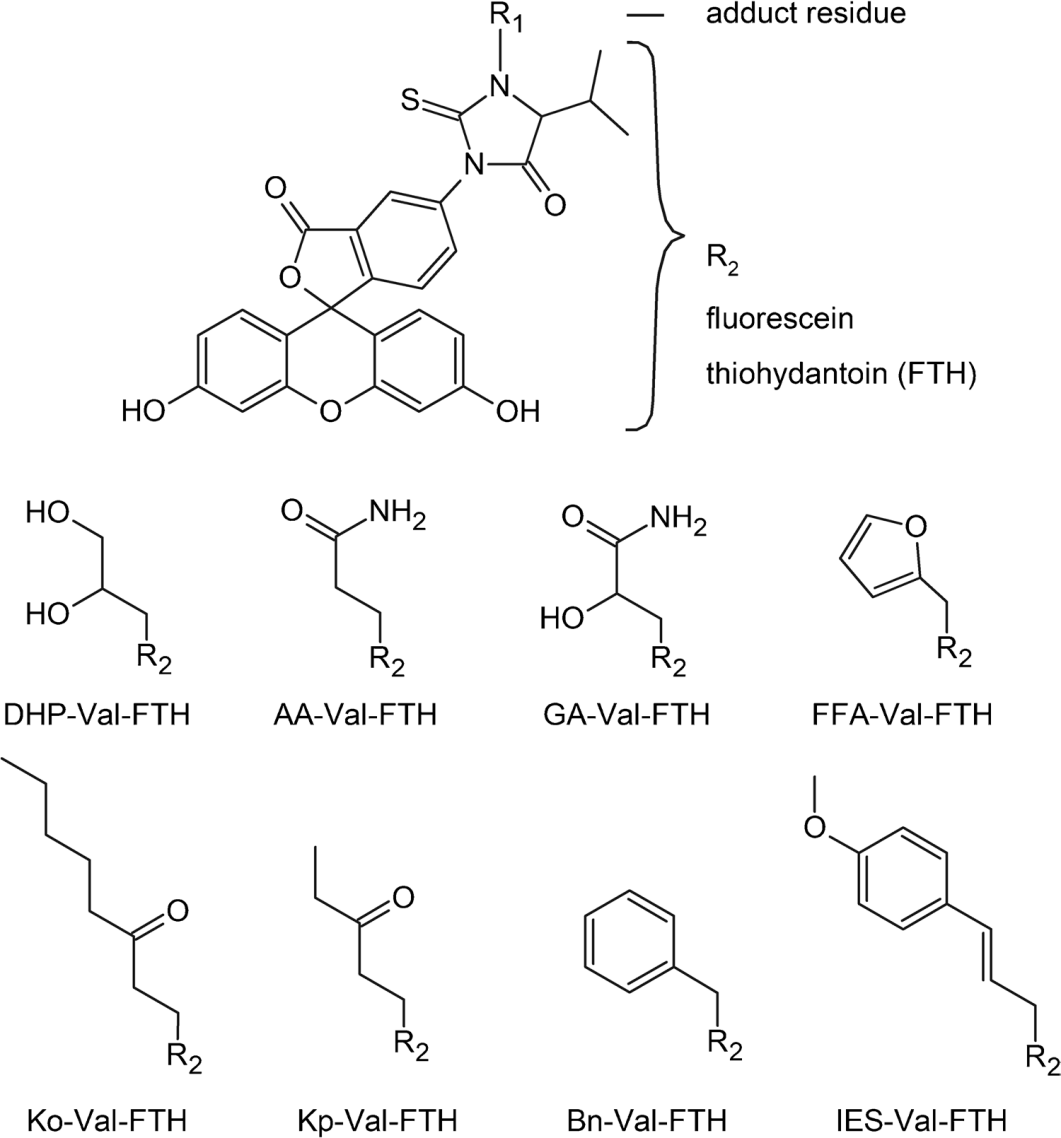
Chemical structures of the FTH analytes released after FITC–mediated cleavage of modified Val residues from the N-termini of hemoglobin

Based on the FI*R*E procedure™, we developed three stand-alone techniques for the specific analyses of Val adducts formed from food carcinogens or their metabolites, i.e., glycidol (*N*-(2,3-dihydroxypropyl)-Val, DHP-Val) [10], furfuryl alcohol (*N*-((furan-2-yl)methyl)-Val, FFA-Val) [17], and estragole as well as anethole (*N*-(*trans*-isoestragole-3′-yl)-Val, IES-Val) [18] following Edman degradation and quantification using isotope-dilution UHPLC-MS/MS and multiple reaction monitoring (MRM). The molecular structures of the analytes are depicted in Fig. 1. The considerable economic effort suggests combining similar analytical methods for different analytes into multimethods in order to improve the cost-effectiveness of the analyses. The aim of the current work was to merge the existing techniques and to incorporate five adducts for the exposure assessment of other compounds with a relevance for food safety, namely acrylamide (*N*-(2-carbamoylethyl)-Val, AA-Val), glycidamide (*N*-(2-carbamoyl-2-hydroxyethyl)-Val, GA-Val) [19], benzyl chloride (*N*-benzyl-Val, Bn-Val) [20], 1-penten-3-one (*N*-(3-ketopentyl)-Val, Kp-Val) [21], and 1-octen-3-one (*N*-(3-ketooctanyl)-Val, Ko-Val) [22]. Molecular structures of adduct precursors are summarized in Fig. S2 of the Supplementary information. As a proof-of-principle study, the resulting method was applied to quantify the Val adduct levels using eight specific isotope-labeled standards in blood samples of six smoking and six non-smoking adults.

## Materials and methods

### Chemicals

Fluorescein-5-isothiocyanate (FITC, isomer I, > 95%), *N*,*N*-dimethylformamide (DMF), dimethyl sulfoxide (DMSO), and formic acid (≥ 96%) were purchased from Sigma-Aldrich (Steinheim, Germany). Potassium hydrogen carbonate, aqueous ammonium (25%), methanol, and acetonitrile (for UHPLC-MS) were provided by Merck (Darmstadt, Germany). Ultrapure water was prepared with a Millipore filtering system (Merck). All solvents were analytical grade. The isotope-labeled standard compound 3-(fluorescein-5-yl)-1-(2,3-dihydroxypropyl)-5-*d*_7_-isopropyl-2-thioxo-4-imidazolidinone (DHP-*d*_7_-Val-FTH) was synthesized as described by Hielscher et al. [23], and 3-(fluorescein-5-yl)-1-(2-carbamoylethyl)-5-*d*_7_-isopropyl-2-thioxo-4-imidazolidinone (AA-*d*_7_-Val-FTH) was synthesized by Dr. Seidel at the Biochemical Institute for Environmental Carcinogens (Grosshansdorf, Germany) [13]. 3-(Fluorescein-5-yl)-1-(2-carbamoyl-2-hydroxyethyl)-5-*d*_7_-isopropyl-2-thioxo-4-imidazolidinone (GA-*d*_7_-Val-FTH), 3-(fluorescein-5-yl)-1-((furan-2-yl)methyl)-5-*d*_7_-isopropyl-2-thioxo-4-imidazolidinone (FFA-*d*_7_-Val-FTH), [^13^C_5_,^15^ N]3-(fluorescein-5-yl)-1-(3-ketopentyl)-5-isopropyl-2-thioxo-4-imidazolidinone ([^13^C_5_,^15^ N]Kp-Val-FTH), [^13^C_5_,^15^ N]3-(fluorescein-5-yl)-1-(3-ketooctanyl)-5-isopropyl-2-thioxo-4-imidazolidinone ([^13^C_5_,^15^ N]Ko-Val-FTH), [^13^C_5_,^15^ N]3-(fluorescein-5-yl)-1-(*trans*-isoestragole-3′-yl)-5-isopropyl-2-thioxo-4-imidazolidinone ([^13^C_5_,^15^ N]IES-Val-FTH), and [^13^C_5_,^15^ N]3-(fluorescein-5-yl)-1-benzyl-5-isopropyl-2-thioxo-4-imidazolidinone ([^13^C_5_,^15^ N]Bn-Val-FTH) were custom synthesized by the ASCA GmbH (Berlin, Germany). The chemical structures of the standard substances showing the stable isotope labels are summarized in Fig. S3, and the purities and the isotopic purities are summarized in Table S1 of the Supplementary information. The dipeptide *N*-(2-carbamoylethyl)-Val-Leu-anilide (AA-VL-An) was purchased from Bachem (Bubendorf, Switzerland) and *N*-(2-carbamoyl-2-hydroxyethyl)-Val-Leu-anilide (GA-VL-An) was a generous gift from Dr. Schettgen (University of Aachen, Germany). The dipeptides *N*-(2,3-dihydroxypropyl)-Val-Leu-anilide (DHP-VL-An) and *N*-((furan-2-yl)methyl)-Val-Leu-OH (FFA-VL) were synthesized as described [10, 17]. The molecular structures of the dipeptides are depicted in Fig. S4 of the Supplementary information. The chemical acronyms defined here were omitted in the abbreviations list.

### Preparation of standard solutions

Stock solutions of the isotope-labeled compounds and the dipeptides were prepared by accurate weighing of 1 mg dry material, which was dissolved in an adequate volume of DMSO to achieve solutions of 5 mmol/L. The working solutions of the isotope-labeled standards (50 nmol/L) and the dipeptides (100 nmol/L) were prepared by dilution in water/acetonitrile (1:1), aliquoted for further use, and stored at – 80 °C.

### Human blood samples

Blood samples were taken from 12 healthy individuals (6 smokers and 6 non-smokers) of the Risks and Benefits of a Vegan Diet (RBVD) study [24]. The study was approved by the ethics committee of the Charité—Universitätsmedizin Berlin (No. EA4/121/16) and performed in accordance with the ethical standards laid down in the 1964 Declaration of Helsinki and its later amendments. All participants got a detailed oral consultation about the rationale of the study and gave informed consent in writing. EDTA tubes S-Monovette® (9 mL, Sarstedt, Numbrecht, Germany) were used for blood drawing. The blood was centrifuged (2500⋅*g*, 12 min) and the plasma was removed. The remaining erythrocytes were washed twice with 0.9% aqueous sodium chloride (2.5 mL) and lyzed by adding 2.5 mL of water. The Hb content determined with a HemoCue Hb 201 + analyzer (Radiometer, Willich, Germany) varied from 11.1 to 14.8 g Hb/dL. The erythrocyte suspensions were stored at − 80 °C until adduct analysis.

### Edman degradation and solid-phase extraction

The technique established in the current study was based on a modified Edman degradation using FITC for the cleavage of the N-terminal valine in Hb developed by Rydberg et al. [12]. Aliquots of the erythrocyte suspensions (containing 35 mg Hb each) were alkalized with 15 μL aqueous potassium hydrogen carbonate (1 M). To each sample, 10 μL of isotope-labeled standard solutions (50 nmol/L) was added for the quantification of the adducts. Samples were mixed and incubated with FITC (5 mg dissolved in 30 μL DMF) for 18 h (37 °C, 800 rpm). Protein and cell debris were precipitated by adding 1.6 mL acetonitrile and samples were centrifuged (18,000**⋅***g*, 10 min). The pH of the supernatant was adjusted with 25 μL aqueous ammonium hydroxide (1 M). The samples were transferred to mixed-mode anion exchange cartridges Oasis MAX (60 mg; Waters, Eschborn, Germany), preconditioned with 2 mL acetonitrile and 2 mL water. The columns were washed with acetonitrile, water, and 1% aqueous formic acid (2 mL each) and the FTH conjugates of Val adducts were eluted twice with 1.5 mL 1% formic acid dissolved in acetonitrile/water (9:1). The combined extracts of each sample were evaporated to dryness under reduced pressure (1 mbar, 20 °C) and reconstituted in 50 μL acetonitrile/water (1:1) containing 1% formic acid.

### UPLC–MS/MS analytical quantification

For the chromatographic separation of the FTH analytes, an Acquity UPLC system (Waters) equipped with an Acquity HSS T3 column (1.8 μm, 2.1 mm × 150 mm, Waters) was used. Samples of 10 μL were injected and eluted with water + 0.1% formic acid (A) and acetonitrile + 0.1% formic acid (B). The following two-step gradient was applied at a flow rate of 0.4 mL/min: 0–1 min (10% B), 1–15 min (10–50% B), 15–21 min (50–70% B), 21–22.5 min (90% B), and 22.5–24 min (10% B). The analytes were detected with a QTrap6500 (Sciex, Darmstadt, Germany) mass spectrometer, equipped with an electrospray ionization source operated in the positive mode. The MRM parameters for the recording of the analytes and the isotope-labeled reference substances are summarized in Table S2 of the Supplementary information. Additional mass spectrometric parameters were as follows: curtain gas, 20 psi; collision-activated-dissociation (CAD) gas, medium; ion source temperature, 450 °C, ion spray voltage, 5500 V; ion source gas 1, 60 psi; and ion source gas 2, 50 psi. The data was recorded and analyzed with Analyst 1.7 Software (Sciex).

The adduct levels were calculated as follows:

$${\mathrm{amount}}_{\mathrm{adduct}}\left[\frac{\mathrm{pmol}}{g\;\mathrm{Hb}}\right]=\frac{\frac{A_{\mathrm{analyte}}}{A_{\mathrm{IS}}}\ast{\mathrm{amount}}_{\mathrm{IS}}\;\left[\mathrm{pmol}\right]}{{\mathrm{amount}}_{\mathrm{Hb}}\;\left[\mathrm g\right]}$$

with *A*_analyte_ and *A*_IS_ as the peak areas of the quantifier signals of the analyte and of the internal standard, respectively, and with amount_IS_ and amount_Hb_ as the quantities of the internal standard and of Hb used for the Edman degradation, respectively.

### Determination of Edman efficiency factors

The adduct levels of AA-Val, GA-VaL, DHP-Val, and FFA-Val were adjusted for the incomplete Val cleavage (“Edman efficiency”). In each sample set, we included ten separate samples of an erythrocyte pool, five of which were spiked with specified amounts of the four dipeptides (AA-VL-An, GA-VL-An, DHP-VL-An, and FFA-VL). The samples were worked up and analyzed as described. After subtracting the background levels of AA-Val, GA-VaL, DHP-Val, and FFA-Val from the adduct levels in the spiked samples, the efficiencies of the Edman degradation reactions were calculated as the ratio from the resulting and the nominal values (= 100%). The ratios reflected the daily Edman efficiencies and were used to correct the levels of the four adducts determined in the human samples [10, 25].

### Validation

The linear range of the quantification method was studied using 16 concentrations between 2.5 pmol/L and 250 nmol/L (DHP-*d*_7_-Val-FTH, AA-*d*_7_-Val-FTH, GA-*d*_7_-Val-FTH), or 15 concentrations between 2.5 pmol/L and 100 nmol/L (FFA-*d*_7_-Val-FTH, [^13^C_5_,^15^ N]Kp-Val-FTH), or 17 concentrations between 0.05 pmol/L and 10 nmol/L ([^13^C_5_,^15^ N]Ko-Val-FTH, [^13^C_5_,^15^ N]IES-Val-FTH, [^13^C_5_,^15^ N]Bn-Val-FTH). The samples were prepared in water/acetonitrile (1:1) acidified with 1% formic acid and in post-processed Edman matrix prepared from about 250 μL human erythrocyte suspension (~ 35 mg Hb per sample from a pool consisting of erythrocytes from four different donors). In the following, the term *Edman matrix* is used to describe the sum of compounds resulting from a FITC–mediated Edman degradation using 35 mg Hb after extraction by Oasis MAX columns as described. The trend lines were calculated by linear regression. The difference of the slopes observed after analyzing the FTH analytes in the presence or absence of Edman matrix was defined as “matrix effect.” The recovery of the sample preparation was determined as the ratio calculated from mass spectrometric peak areas detected for erythrocyte samples (*n* = 6; pool of four different donors) worked up by Edman degradation and then spiked with 20 μL of each analyte solution (50 nmol/L) and for those erythrocyte samples (*n* = 6) that were spiked first and subjected to the Edman degradation afterwards. The limits of detection (LOD) and the limits of quantification (LOQ) were defined by the signal-to-noise ratios (*S*/*N*) with minimum values of *S*/*N* ≥ 3 for the LOD and *S*/*N* ≥ 10 for the LOQ. The intraday precision was calculated as follows. Hb samples were spiked with three concentrations of each of the analytes (LOQ, 5 × LOQ and 25 × LOQ; *n* = 6 each), worked up as described, and analyzed immediately by UHPLC-MS/MS. The interday precision from the analyses was determined with the same sample sets (three concentration levels; *n* = 1) prepared in different weeks (*n* = 5).

### Statistics

Statistical analyses were conducted with SigmaPlot version 14.0 (Systat Software, Inc., Erkrath, Germany). Unless stated otherwise, adduct levels in the blood samples of 12 study participants and data from other studies were presented as median values followed by the range in brackets. Differences between adduct levels in non-smokers and smokers were analyzed with the Mann–Whitney rank-sum test. Differences with *p* values < 0.05 were considered statistically significant.

## Results and discussion

### Development of the method

Five of the eight analytes (AA-Val, GA-Val, Bn-Val, Ko-Val, and Kp-Val) were analyzed in our laboratory for the first time. The FTH conjugates of these adducts were synthesized for the unambiguous identification of the respective adducts detected in Hb samples. In addition, isotope-labeled standards were prepared for the sake of quantification. Starting from the basic techniques reported for single adducts [10, 17, 18], several adaption steps were taken for the simultaneous determination of all eight analytes (the Edman degradation was used as described without any changes). The most important changes were as follows. Firstly, the chromatographic conditions were altered. Initial analyses showed that the Edman matrix produced a relatively dense chromatographic background, which, especially in the case of the analytes AA-Val-FTH, GA-Val-FTH, and DHP-Val-FTH, interfered with the specificity of mass spectrometric detection. Improving the chromatography conditions for a better separation of the analytes from the remnants of the Edman degradation and from each other led to the choice of an Acquity HSS T3 column (1.8 μm, 2.1 mm × 150 mm) and a relatively long two-step gradient of 21.5-min duration changing from 10 to 90% eluent B (acetonitrile and 0.1% formic acid). Secondly, we paid special attention to the choice of quantifier and qualifier transitions (summarized in Table S2 of the Supplementary information). An essential criterion for the specificity of detection is that the quantifier signals of the analytes and the respective isotope-labeled internal standards were free of interferences from contaminants of the Edman matrix. To select appropriate quantifier peaks, we compared the peak area ratios of all MRM transitions from the analysis of a particular FTH analyte and its respective isotope-labeled standard in the presence of the Edman matrix with those observed after direct injection. Equivalent peak area ratios under different conditions (summarized in Table S3 of the Supplementary information) confirmed the specificity of detection of quantifier/qualifier signals.

### Validation

The method was validated using the isotope-labeled standards of the FTH analytes because the common presence of the Hb adducts in human blood samples hindered the validation with unlabeled substances. The linearity of the detection range and the LOD and LOQ values were determined with dilution series of the isotope-labeled FTH analytes dissolved in pure solvents or in the presence of the Edman matrix (Fig. S5 of the Supplementary information). The analyte concentration ranges with a linear response (usually between two and three orders of magnitude) and the LOQ values (between 0.014 and 3.6 pmol/g Hb) are summarized in Table 1. Comparing the slopes of the linear regressions of the dilution series injected either directly or in the presence of the Edman matrix allowed estimating the matrix effect for each analyte. Practically no effect of the Edman matrix was observed for [^13^C_5_,^15^ N]Ko-Val-FTH and [^13^C_5_,^15^ N]IES-Val-FTH. Intermediate effects (22.1 to 53.2%) were observed for most of the FTH analytes. The strong matrix effect in case of AA-Val-FTH detection (87.1%) indicated the presence of co-eluting compounds in the sample interfering with analyte ionization. In contrast to the matrix effects, the recoveries of the SPE (53.9 to 68.9%) were similar for all analytes, which is in line with the high structural similarity of the FTH conjugates.

**Table 1:** Validation parameters for the analyses of FTH conjugates of modified Val residues cleaved by FITC from the N-termini of Hb

**Analyte**	**Recovery**^a^	**Linear range**^b^	**LOQ**	**LOQ**^c^	**Matrix effect**
	**%**	**nmol/L**	**nmol/L**	**pmol/g Hb**	**%**
AA-*d*_7_-Val-FTH	65.4 ± 13.0	0.25–250	0.5	0.71	87.1
GA-*d*_7_-Val-FTH	67.4 ± 6.3	0.25–250	0.5	0.71	53.2
DHP-*d*_7_-Val-FTH	68.9 ± 7.7	0.25–250	0.5	0.71	44.5
FFA-*d*_7_-Val-FTH	53.9 ± 4.5	1–100	2.5	3.6	32.9
[^13^C_5_,^15^ N]IES-Val-FTH	66.0 ± 8.5	0.005–10	0.01	0.014	1.8
[^13^C_5_,^15^ N]Bn-Val-FTH	60.7 ± 3.6	0.01–10	0.025	0.036	22.1
[^13^C_5_,^15^ N]Ko-Val-FTH	67.0 ± 6.0	0.01–10	0.025	0.036	− 1.0
[^13^C_5_,^15^ N]Kp-Val-FTH	67.8 ± 6.6	0.025–100	0.05	0.071	36.5

Due to the background of the adducts in human erythrocyte samples, the validation parameters were determined using the respective isotope-labeled compounds

^a^Recovery of the sample preparation; mean values and SD of six samples

^b^The lower limit of the linear range marks the limit of detection (LOD)

^c^The LOQ (pmol/g Hb) was calculated with the standard parameters of 35 mg Hb used for the Edman degradation and a final sample volume of 50 μL

**Fig. 2:**
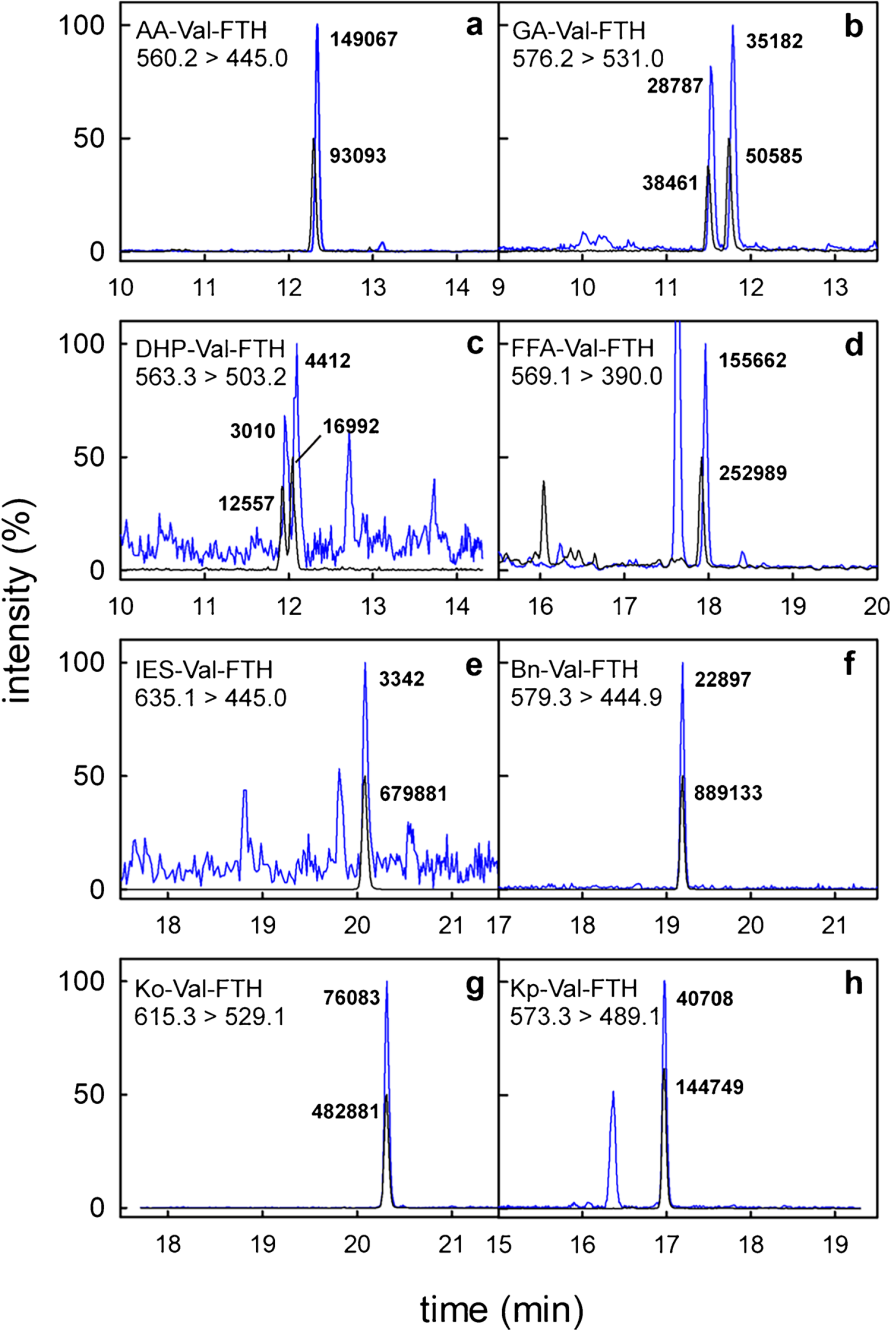
UHPLC-MS/MS chromatograms of the quantifier traces (blue lines) from an Edman degradation sample containing AA-Val-FTH (a, *m*/*z* 560.2 → 445.0), GA-Val-FTH (b, *m*/*z* 576.2 → 531.0), DHP-Val-FTH (c, *m*/*z* 563.3 → 503.2), FFA-Val-FTH (d, *m*/*z* 569.1 → 390.0), IES-Val-FTH (e, *m*/*z* 635.1 → 445.0), Bn-Val-FTH (f, *m*/*z* 579.3 → 449.9), Ko-Val-FTH (g, *m*/*z* 615.3 → 529.1), and Kp-Val-FTH (h, *m*/*z* 573.3 → 489.1), which were normalized to 100% signal intensity. The transitions of the respective isotope-labeled standard compounds (black lines) were adjusted to 50% signal intensity. Absolute intensities of analyte peaks and standard compounds are given. The peak areas correspond to nominal concentrations of 10 nmol/L for all isotope-labeled standard compounds, i.e., 0.1 pmol injected on-column. The chromatographic differentiation of GA-Val-FTH (panel b) and DHP-Val-FTH (panel c) into two peaks reflects the formation of two diastereomers from the nucleophilic attack of valine nitrogens at the terminal carbons of the epoxide rings of glycidamide or glycidol, respectively [23]

The analytes were divided into two groups regarding the LOQ (Table 1), with lower values (0.014–0.071 pmol/g Hb) observed for the analyses of [^13^C_5_,^15^ N]IES-Val-FTH, [^13^C_5_,^15^ N]Bn-Val-FTH, [^13^C_5_,^15^ N]Kp-Val-FTH, and [^13^C_5_,^15^ N]Ko-Val-FTH, which took advantage of two main factors. First, the analytes were well separated (retention times between 17.0 and 20.3 min, Fig. 2) from more hydrophilic byproducts of the FITC–mediated Edman degradation, which minimized the matrix effect. Also, the elution with a higher acetonitrile content was shown to improve the ionization, which increased the sensitivity of mass spectrometric detection [26]. Second, the MRM fragmentation chromatograms of these analytes with relatively high molecular masses (*M*_W_ = 578.6–640.7 g/mol) were less impaired by the background noise level compared to those with lower molecular weight, AA-*d*_7_-Val-FTH, GA-*d*_7_-Val-FTH, DHP-*d*_7_-Val-FTH, and FFA-*d*_7_-Val-FTH (*M*_W_ = 566.6–582.6 g/mol). In these cases of usually more hydrophilic compounds, the co-elution of the Edman matrix produced by FITC led to high noise levels and higher LOQ values.

The LOQ of the techniques focused on single analytes (FFA-Val: 0.9 pmol/g Hb [17], DHP-Val: 1.4 pmol/g Hb [23], IES-Val: 0.0023 pmol/g Hb [18]) and the LOQ values of the current method were in the same range. It must be noted that the LOQ for FFA-*d*_7_-Val-FTH determined here was greatly increased due to the high noise level in the trace *m/z* 576.1 → 390.0 (Fig. 2). In contrast, the trace for the quantification of FFA-Val-FTH (*m*/*z* 569.1 → 390.0) was relatively free of interferences. Estimating the LOQ from the *S*/*N* of all 24 FFA-Val-FTH analyses conducted in the current study (described in chapter 3.3.) by linear extrapolation led to a value of 0.9 pmol/g Hb, confirming the LOQ previously reported [17, 27].

Inter- and intraday precisions for all eight analytes are summarized in Table 2. The average CV (%) of the intraday precision over all analytes at the LOQ, 5 × LOQ, and 25 × LOQ were 9.7 ± 3.8, 7.3 ± 1.8, and 7.0 ± 2.6, respectively. The average CV (%) of the interday precision at the LOQ, 5 × LOQ, and 25 × LOQ were 9.8 ± 2.2, 7.0 ± 3.4, and 6.0 ± 1.7, respectively. The individual values meet the criteria for the inter- and intraday precision of biomarker analyses by chromatographic assays (not to exceed ± 15%, except ± 20% at the LOQ) stated by the Food and Drug Administration [28].

**Table 2:** CV (%) of five (interday precision) or six (intraday precision) independent analyses at three different concentration levels, corresponding to the respective LOQ, 5 × LOQ, and 25 × LOQ

Analyte	Intraday			Interday		
	**LOQ**	**5 × LOQ**	**25 × LOQ**	**LOQ**	**5 × LOQ**	**25 × LOQ**
AA-*d*_7_-Val-FTH	17.4	8.1	7.2	12.7	3.0	4.2
GA-*d*_7_-Val-FTH	9.0	10.3	9.8	11.9	13.9	8.6
DHP-*d*_7_-Val-FTH	9.1	5.3	9.0	6.4	10.2	8.7
FFA-*d*_7_-Val-FTH	4.2	4.7	9.2	11.1	5.7	5.6
[^13^C_5_,^15^ N]IES-Val-FTH	12.9	9.5	6.5	6.7	8.4	5.8
[^13^C_5_,^15^ N]Bn-Val-FTH	10.3	7.1	7.6	10.8	5.6	5.1
[^13^C_5_,^15^ N]Ko-Val-FTH	6.5	6.8	5.4	8.9	3.4	4.1
[^13^C_5_,^15^ N]Kp-Val-FTH	8.2	6.6	1.2	9.9	5.7	5.9

Due to the presence of background levels in human Hb, the analyses were performed with the isotope-labeled compounds

### Adduct quantification in human erythrocytes

In order to show the applicability, the technique was used to quantify the Val adducts in Hb samples of twelve adults (six non-smokers, six smokers). Figure 2 shows a complete set of analytical results in a sample from a non-smoking adult with the quantifier traces of the analytes together with the signals of the isotope-labeled standard compounds. It was an interesting detail to observe that all the deuterated standards preceded the analyte peaks by about 3 s, whereas the ^13^C- and ^14^ N-labeled standards showed the same retention times as the non-labeled analytes. The adduct levels of individual study participants are summarized in Table S4 of the Supplementary information. Seven out of eight analytes were well quantifiable in all samples. Only the signals of IES-Val were usually in the range between LOD and LOQ.

**Fig. 3:**
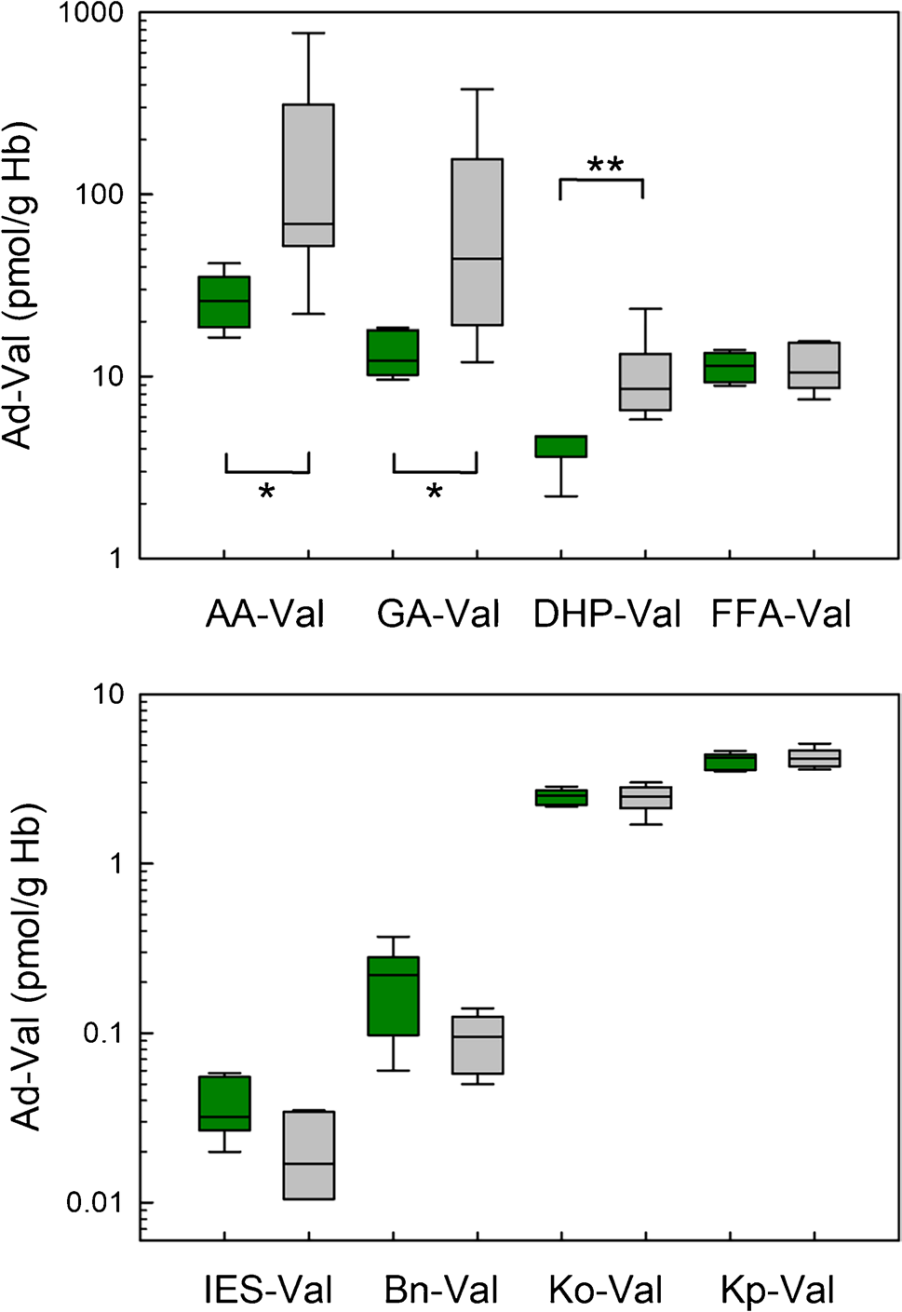
Levels of different Val adducts in blood samples of 12 adults of the RBVD study (data from 6 non-smokers and 6 smokers are represented by green boxes and gray boxes, respectively). The upper panel shows the adducts of common heat-induced contaminants AA-Val, GA-Val, DHP-Val, and FFA-Val, and the lower panel shows the adducts of IES-Val, Bn-Val, Ko-Val, and Kp-Val. Lines and boxes represent median values and the lower and upper quartiles, respectively, and the error bars represent the 10th and 90th percentiles. The levels of AA-Val, GA-Val, and DHP-Val from non-smokers and smokers were significantly different (* *p* < 0.05, ** *p* < 0.005, Mann–Whitney rank-sum test)

The influence of smoking on the exposure is presented as a bar chart (Fig. 3). In addition, the median as well as the minimum and maximum values of adduct levels in non-smokers and smokers are summarized in Table 3. The levels of AA-Val, GA-Val, and DHP-Val were significantly higher in smokers (*p* = 0.015, 0.015, and 0.002, respectively) compared to those in non-smokers. In contrast, there were no differences between levels of FFA-Val, Bn-Val, IES-Val, Ko-Val, and Kp-Val in blood samples from non-smokers and smokers. The data indicated that there is a tobacco-smoke-related exposure to acrylamide, glycidamide, and glycidol, which are also common heat-induced contaminants in foodstuffs. Generalizing conclusions are not permitted based on these results; the group of study participants is too small, and in the case of the smokers, there were no data reported on the number of cigarettes smoked per day.

**Table 3:** Median levels of the adducts (pmol/g Hb) as well as minimum and maximum values in brackets in erythrocyte samples of human study participants of the current study in comparison to data from previous studies

Adduct^a^	Source	Adduct level, **this study** pmol/g Hb		Adduct levels, **other studies** pmol/g Hb				Ref
		**Non-smokers**	**Smokers**	**Non-smoker**	***n***	**Smokers**	***n***	
AA-Val	Acrylamide	25.9 (16.4–41.9)	69.0 (22.0–770)	39.6 (14.7–92.4)	30	137 (32.1–371)	30	[29]^b,c^
GA-Val	Glycidamide	12.2 (9.6–18.5)	44.2 (12.0–378)	34.1 (11.2–96.6)	30	96.1 (25.4–247)	30	[29]^b,c^
DHP-Val	Glycidol	4.7 (2.2–4.7)	8.6 (5.8–23.5)	6.2 (1.4–16)	42			[34]^c^
				4.0 (3.2–5.1)	11			[10]
				10.3 (6.3–14.0)	6	23.4 (18.1–31.4)	6	[35]
FFA-Val	Furfuryl alcohol	11.5 (8.9–14.0)	10.5 (7.5–15.6)	14.1 (8.2–26.3)	59	17 (11.2–21.4)	13	[27]
IES-Val	Estragole	0.032 (0.02–0.058)	0.017 (< LOQ–0.034)	0.016 (0.012–0.047)	14	–		[18]
Bn-Val	Benzyl chloride	0.22 (0.06–0.37)	0.09 (0.05–0.14)	–		–		
Ko-Val	1-Octen-3-one	2.52 (2.17–2.84)	2.48 (1.7–3.01)	62 ± 15 (44–91)^d^			12	[22]
Kp-Val	1-Penten-3-one	4.21 (3.49–4.62)	4.15 (3.59–5.11)	43 ± 14 (26–68)^e^	6	38 ± 10 (25–53)^e^	6	[21]

^a^The adducts may not be formed exclusively from the depicted precursors, e.g., IES-Val is formed from estragole and *trans*-anethole, and Bn-Val, although discussed primarily in relation to benzyl chloride exposure, may also be formed from benzyl bromide or benzaldehyde

^b^From the multitude of data on AA-Val and GA-Val, we selected an exemplary study with German participants

^c^The data was reported in pmol/g globin

^d^Adduct levels were estimated using a calibration curve with peak area ratios of AA-Val-FTH/AA-d_7_-Val-FTH. There was no significant difference between the adduct levels in blood samples from non-smoker and smokers (each *n* = 6)

^e^Values were reported as mean ± SD

The current data was compared with results from previous analyses by other work groups (Table 3). For this sake, the molecular weight difference between globin (62 kDa) used for a classic Edman degradation in older studies [8] and Hb (64.5 kDa) used for the FITC–mediated Edman degradation is not relevant. In the cases of AA-Val, GA-Val, DHP-Val, FFA-Val, and IES-Val, the adduct levels detected in the current study were in the same order of magnitude compared to those observed in previous studies (Table 3).

The quantification of FFA-Val indicated that changes of the analytical parameters had an effect on the results. Using the current multimethod, the repeated analysis of 12 samples from the RBVD study resulted in lower FFA-Val levels compared to those reported before (average deviation from the mean 20.7%) [27]. The results of two FFA-Val quantification experiments using the former method and the current one described here indicated two reasons for the deviation (described in the Supplementary information). First, the use of cyanoacetic acid in the previous work led to an approximate 10% increase of FFA-Val levels, probably resulting from a contamination co-eluting with the quantifier trace (*m*/*z* 569.1 → 390.0). (The other major difference between the techniques was a change in the chromatographic conditions, using an HSS T3 column (Waters) in the current study instead of a Hypersil GOLD column (Thermo) as described by Monien et al. [27] (FFA-Val experiment S1 in the Supplementary information). This had no effect on the resulting FFA-Val levels.) Second, FFA-Val in the erythrocyte samples may be unstable (FFA-Val experiment S2 in the Supplementary information). The stability within a timeframe of 2 years was tested by repeating the analyses of FFA-Val in 12 Hb samples from the RBVD study using the previous conditions [27]. The FFA-Val levels were on average 9% lower compared to those determined 26 months ago, indicating that the adduct FFA-Val may not be stable even during storage at − 80 °C. The decay, however, is relatively slow, so we cannot further prove this hypothesis with a short-term experiment. It is of note that the levels of DHP-Val determined in the current work, which was also analyzed 30 months ago in the same samples (manuscript in preparation), were well reproducible (average deviation from the mean 6.9%). Hence, the difference of FFA-Val levels is not due to an inhomogeneity or a general aging process of the erythrocyte samples.

The Hb adducts Ko-Val and Kp-Val (Fig. 1) were previously detected by Carlsson et al. at much higher levels as compared to the actual data (Table 3). The reason for that is unclear. Due to the suspected instability of the adducts, we avoided painstakingly elevated temperatures and prolonged waiting times during erythrocyte sample preparation. The sample processing was essentially the same as reported by Carlsson et al. [21]. It is of note that the reliability of our method is underlined by the use of authentic isotope-labeled standards for the quantification of Ko-Val and Kp-Val which were not available to Carlsson et al.

### Strengths and limitations of the current technique

Various advantages of the FITC–mediated Edman degradation were described by von Stedingk et al., e.g., the facile synthesis of the standard compounds for the FTH analytes in two-step reactions, the specificity of mass spectrometric detection due to the multitude of fragmentations, and the possibility of using erythrocytes and even whole blood samples instead of globin for the analysis [13].

A fundamental problem of the quantification of Hb adducts is that the fraction of the Val residues cleaved by the Edman degradation (the so-called Edman efficiency) remains unknown, which leads to a method-inherent underestimation of Hb adduct levels. Unfortunately, there is no reference material available, i.e., erythrocytes with defined levels of certain adducts, in order to control the accuracy of the technique. To improve the accuracy, chemically synthesized peptides with adducted valine residues at the N-terminus were used to determine the Edman reaction turnover in separate samples. In our recent works, we determined the Edman efficiency by analyzing adducted dipeptides (*N*-R_adduct_-Val-Leu-anilide) assuming that the yields of the FITC–mediated cleavage of modified Val residues from Hb or from the dipeptide were similar. In the case of the dipeptide DHP-VL-An, for example, the average Edman efficiency in nine independent experiments was between 67.0 and 85.3% (mean ± SD = 77.7 ± 4.3%) [25]. Single values were applied to correct DHP-Val levels determined in the set of regular erythrocyte samples analyzed on the same day. It is a benefit of the current method that we incorporated dipeptides for the correction of AA-Val, GA-Val, DHP-Val, and FFA-Val levels. The Edman efficiency was not determined for the other adducts. We synthesized the dipeptide IES-VL-OH and IES-VL-An; however, the hydrophobicity of the compounds precluded the dissolution in the Edman sample (data not shown). The other adducts considered in the current study (Bn-Val, Ko-Val, and Kp-Val) are still on an investigational level, and therefore, the experimental effort was not invested. In these cases, it was assumed that the Edman degradation was complete. It is of note that the current approach to determine the Edman efficiency can be further improved. Vesper et al. used adducts of isotope-labeled octapeptides AA-Val(^13^C_5_^15^N)-HLTPEEK and GA-Val(^13^C_5_^15^N)-HLTPEEK for the quantification of AA-Val and GA-Val. They mimic Hb probably better than the dipeptides and the incorporation of isotope-labeled valine residues makes the determination of the Edman efficiency in a separate sample set obsolete [29]. However, the custom synthesis of one of the octapeptides costed about 30,000 $ (H. Vesper, personal communication), which is too expensive for most laboratories if multiple adducts are targeted.

Two aspects can be considered as limitations of the technique. First, the matrix resulting from FITC–mediated Edman degradations produces a relatively dense background, which co-elutes with AA-Val, GA-Val, and DHP-Val. This affects LOQ values and made the choice of quantifier traces without interferences for these analytes and their isotope-labeled standards difficult. It may be advantageous to use a high-resolution mass spectrometer, e.g., an Orbitrap (Thermo), for the analyses, which would greatly increase the specificity of detection. Second, with the targeted technique presented here, unknown adducts remain undetected. Carlsson and Törnqvist adopted a strategy for the detection and identification of unknown adducts in Hb, a so-called adductomics approach [16, 30]. The accuracy of quantification may be limited. Nevertheless, the recording of all unknown adducts in a sample bears a lot of potential for the future use in human biomonitoring [31].

## Conclusions

The Hb adduct quantification on the basis of the FITC–mediated Edman degradation was introduced by Rydberg et al. more than a decade ago [12]. The principle was used in the current work to devise an isotope-dilution technique for the simultaneous quantification of eight different Hb adducts. The chromatographic conditions employed for the multimethod led to an improved detection of DHP-Val-FTH [23] but to a slight loss of sensitivity in the case of IES-Val-FTH [18]. The advantages of simultaneous analysis outweigh the small loss in sensitivity of individual analytes. Efficient isotope-dilution techniques have been established for the other five analytes, of which Bn-Val, Ko-Val, and Kp-Val have not been studied extensively. In summary, the LOQs, linear ranges, and precision values of the analyses were satisfactory [28].

Three advantages of the current method stand out in comparison to previous works. First, the method is convenient and simple in comparison to Edman degradation analyses using globin chains, which are tedious to isolate from Hb samples [15, 32, 33]. Second, the accuracies of quantification are supported by the integration of an individual isotope-labeled standard for each of the adduct analytes. This renders obsolete the error-prone analysis using multiple external calibration solutions. And third, the accuracies were increased further for the quantification of AA-Val, GA-Val, DHP-Val, and FFA-Val due to the application of the respective dipeptide standards that allows for the correction of the adduct levels with the efficiency of the Edman degradation.

As a proof of principle, the eight adducts were quantified in 12 samples of human adults in the RBVD study. Three of the adducts quantified here (AA-Val, GA-Val, and DHP-Val) were well studied in different populations, whereas corresponding data on the other five adducts are scarce (FFA-Val, IES-Val, Bn-Val, Ko-Val, and Kp-Val). The median levels of AA-Val, GA-Val, and DHP-Val were in the same range as in previous studies, and the elevated adduct levels in smokers showed that acrylamide, glycidamide, and glycidol are present in tobacco smoke. The significance of the relatively low levels of Bn-Val and the medium levels detected in the cases of Ko-Val and Kp-Val is not yet clear.

In the future, we will apply the technique to the analysis of the internal exposure in ongoing epidemiological studies involving omnivores, vegans, and strict raw food eaters (not consuming any food heated to temperatures higher than 42 °C).

## Supplementary Information

Below is the link to the electronic supplementary material.

Supplementary file1 (DOCX 984 KB)

## Abbreviations

AA-Val: *N*-(2-Carbamoylethyl)-Val
Bn-Val: *N*-Benzyl-Val
DHP-Val: *N*-(2,3-Dihydroxypropyl)-Val
EFSA: European Food Safety Authority
FFA-Val: *N*-((Furan-2-yl)methyl)-Val
FITC: Fluorescein-5-isothiocyanate
FTH: Fluorescein thiohydantoin
GA-Val: *N*-(2-Carbamoyl-2-hydroxyethyl)-Val
Hb: Hemoglobin
IES-Val: *N*-(*trans*-Isoestragole-3′-yl)-Val
Ko-Val: *N*-(3-Ketooctanyl)-Val
Kp-Val: *N*-(3-Ketopentyl)-Val
LOD: Limit of detection
LOQ: Limit of quantification
MRM: Multiple reaction monitoring
RBVD study: Risks and Benefits of a Vegan Diet study
*S*/*N*: Signal-to-noise ratio
SPE: Solid-phase extraction
