# Supplementary material for: Simultaneous quantification of eight hemoglobin adducts of genotoxic substances by isotope-dilution UHPLC-MS/MS
Source: Anal Bioanal Chem. 2022 Jun 2;414(19):5805–15. doi: 10.1007/s00216-022-04143-y (PMC9293867; doi:10.1007/s00216-022-04143-y)
Supplement: Supplementary file 1 — Supplementary file1 (DOCX 984 KB) [file 216_2022_4143_MOESM1_ESM.docx]

**Simultaneous quantification of eight hemoglobin adducts of genotoxic substances by isotope-dilution UHPLC-MS/MS**

Fabian Gauch, Klaus Abraham, Bernhard H. Monien

***Supplementary Information***

**
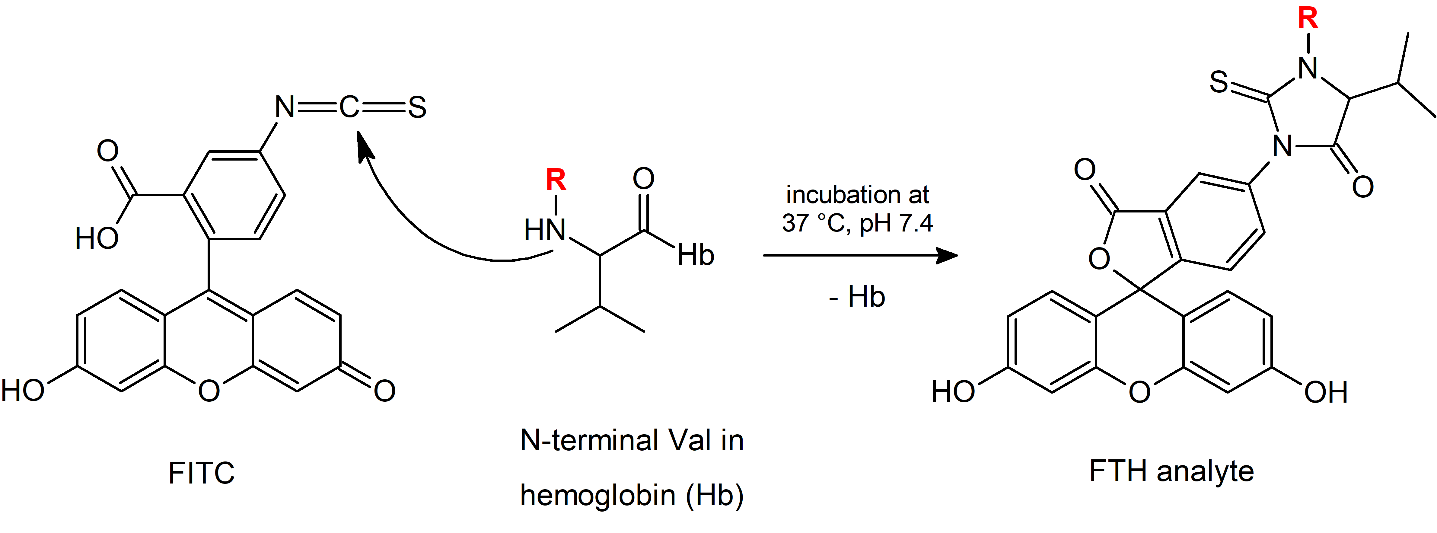
**

**Fig. S1** The Edman degradation with FITC leads to formation of a fluorescein thiohydantoin (FTH) conjugate. The FTH analyte is specific for the Val modified with a reactive electrophile (R).


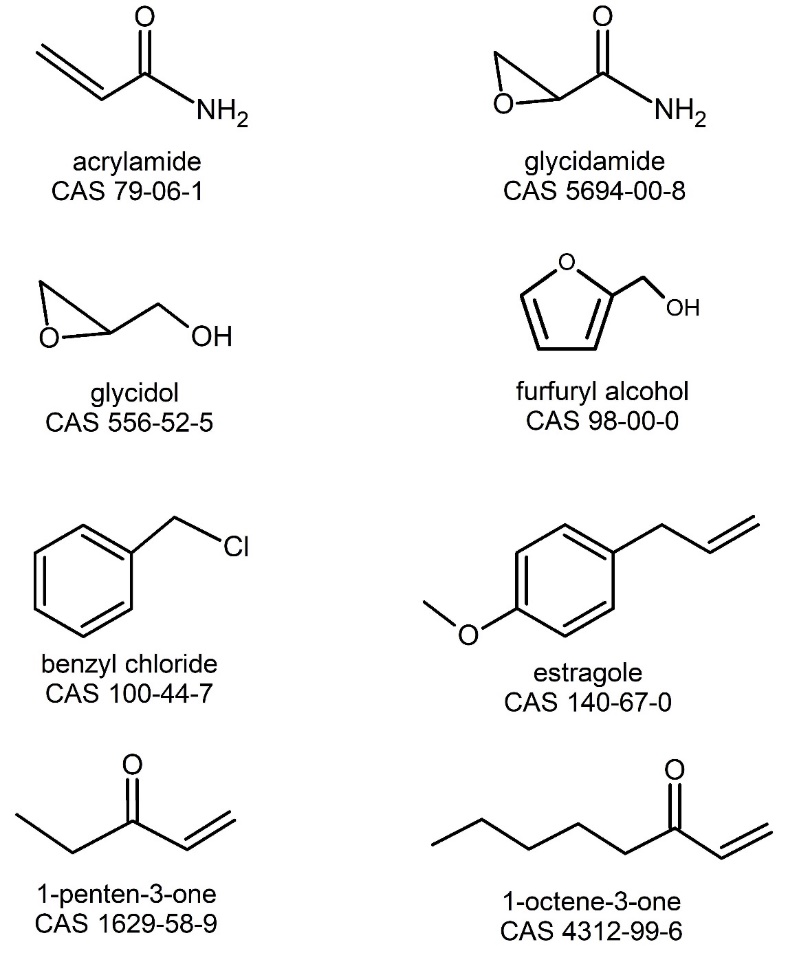


**Fig. S2** Molecular structures of adduct precursors as described in the introduction. Some of the adducts may not be formed exclusively from the depicted precursors. For example, the adduct IES-Val in Hb is formed from estragole and *trans*-anethole [1], and Bn-Val, although discussed primarily in relation to benzyl chloride exposure, may also be formed from benzyl bromide or benzaldehyde [2]. In theses cases, Fig. S2 depicts the most important contributors according to current literature.


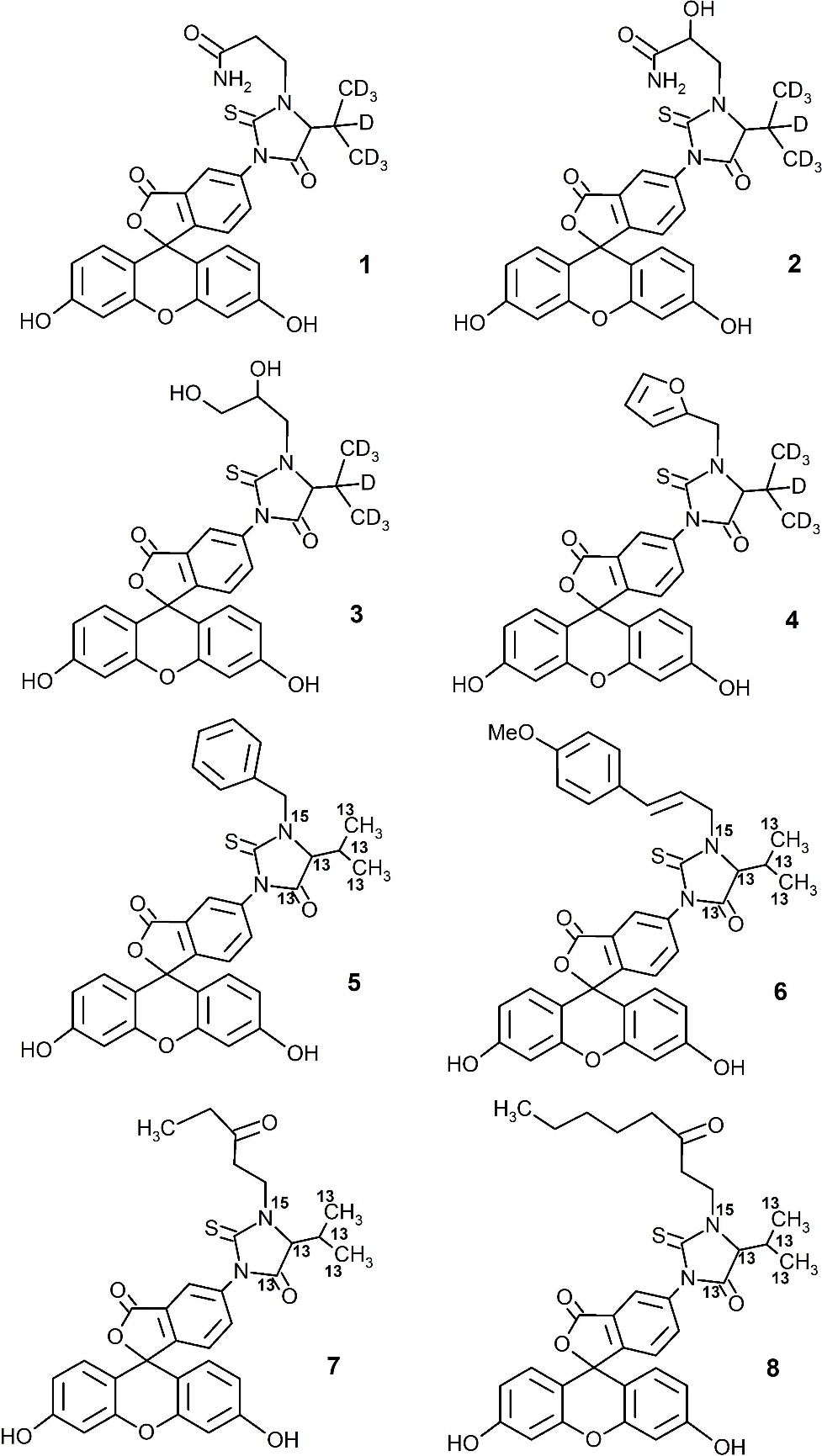


**Fig. S3** Molecular structures of stable isotope-labeled standard substances used for quantification of Hb adducts. The substances AA-d_7_-Val-FTH (**1**), GA-d_7_-Val-FTH (**2**), DHP-d_7_-Val-FTH (**3**) and FFA-d_7_-Val-FTH (**4**) were deuterated at the Val side chain. The compounds [^13^C_5_,^15^N]Bn-Val-FTH (**5**), [^13^C_5_,^15^N]IES-Val-FTH (**6**), [^13^C_5_,^15^N]Kp-Val-FTH (**7**) and [^13^C_5_,^15^N]Ko-Val-FTH (**8**) contained ^13^C and ^14^N at the marked positions.


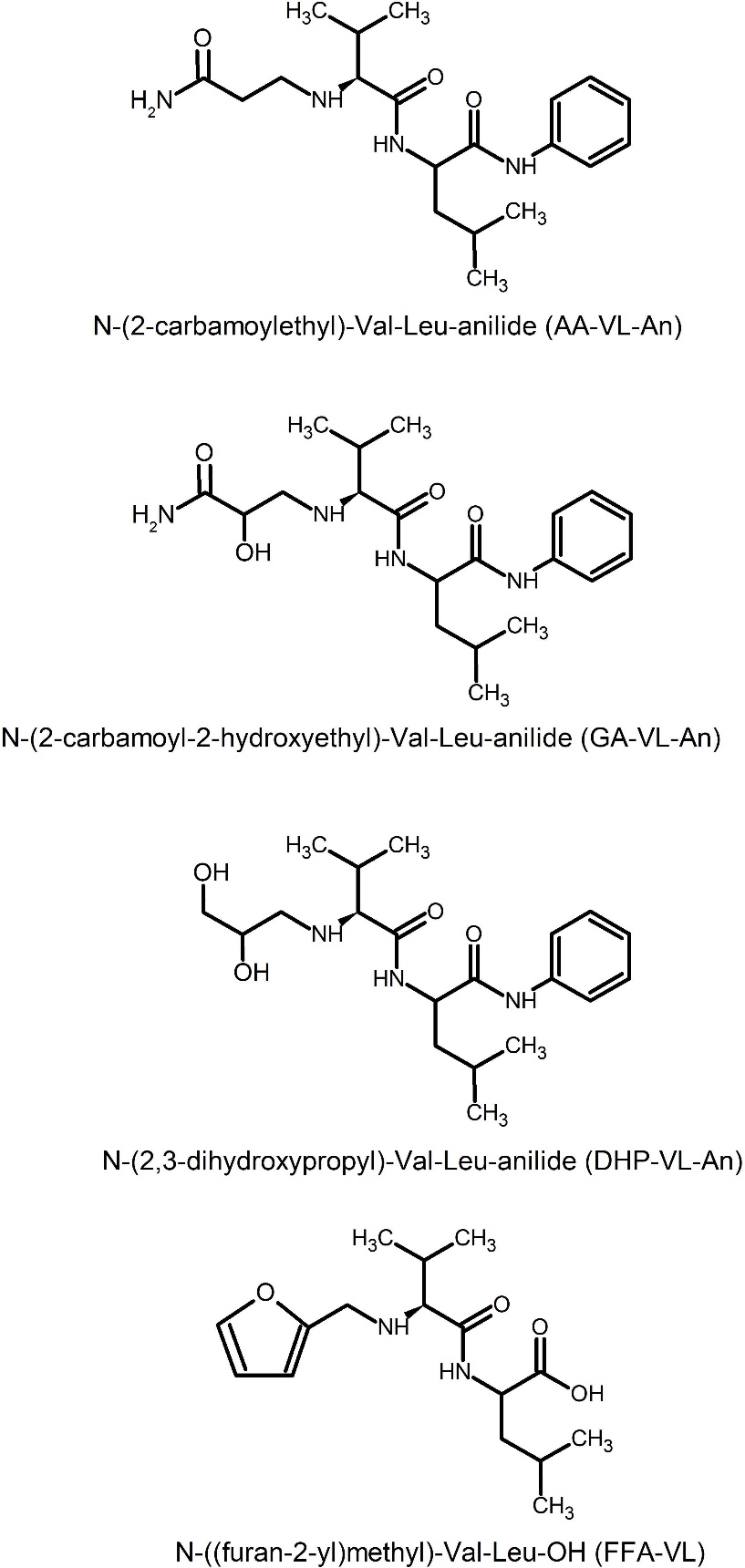


**Fig. S4** Molecular structures of the dipeptides used for determining the efficiency of the Edman degradation.


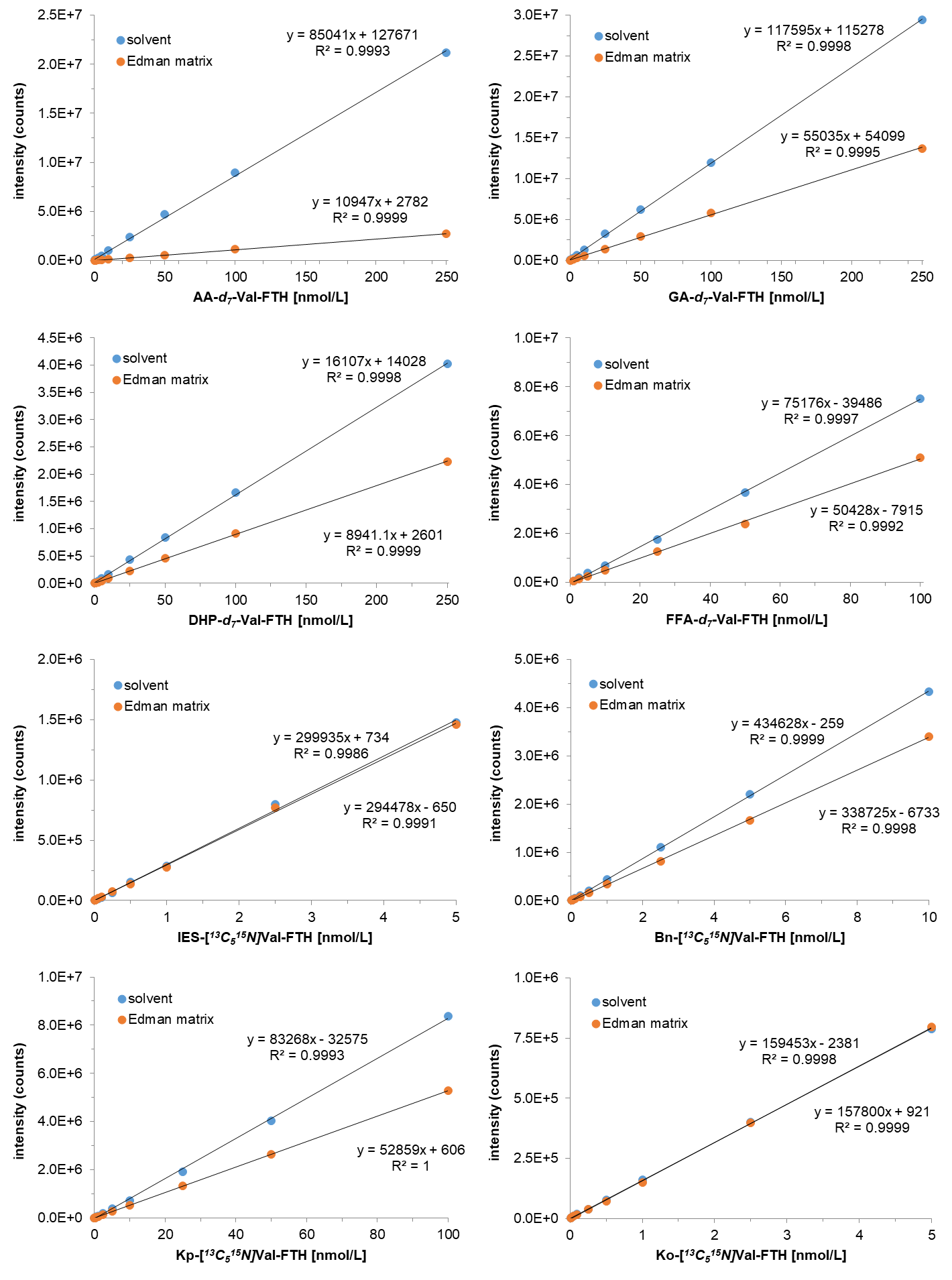


**Fig. S5** The linearity of detection was determined using 15 to 17 solutions of the isotope-labeled standards, which were directly injected without biomatrix (blue dots) or in the presence of processed erythrocyte samples (´Edman matrix´, red dots). The data were fitted with a trend line by linear regression (R^2^ ≥ 0.999). The matrix-dependent reduction of the mass spectrometric signals estimated from the difference of the slopes is summarized in Table 1.

| **Table S1** Chemcial purities and isotopic purities of the standard compounds used in the current study. | | | |
| --- | --- | --- | --- |
| **adduct** | **supplier** | **chemical purity*^a^*** | **isotopic purity*^b^*** |
| AA-d_7_-Val-FTH | Biochemical Institute for Environmental Carcinogens (Grosshansdorf, Germany) | > 99% | > 98% |
| GA-d_7_-Val-FTH | ASCA GmbH (Berlin, Germany) | 98.7% | 99.3% (NMR) |
| DHP-d_7_-Val-FTH | Biochemical Institute for Environmental Carcinogens (Grosshansdorf, Germany) | > 99% | > 98% |
| FFA-d_7_-Val-FTH | ASCA GmbH (Berlin, Germany) | 98.4% | 99.7% (NMR) |
| [^13^C_5_,^15^N]IES-Val-FTH | ASCA GmbH (Berlin, Germany) | 98.6% | 98% ^15^N, 98% ^13^C |
| [^13^C_5_,^15^N]Bn-Val-FTH | ASCA GmbH (Berlin, Germany) | 99.2% | 98% ^15^N, 98% ^13^C |
| [^13^C_5_,^15^N]Kp-Val-FTH | ASCA GmbH (Berlin, Germany) | 97.6% | 98% ^15^N, 98% ^13^C |
| [^13^C_5_,^15^N]Ko-Val-FTH | ASCA GmbH (Berlin, Germany) | 98.6% | 98% ^15^N, 98% ^13^C |
| *a* The chemical purities, as reported from the suppliers, were determined by HPLC-UV at wavelengths between 230 nm and 274 nm.  *b* The isotopic purities are from the certificates of analysis of the isotope-labeled valine (L-valine-d_8_ or L-valine-^13^C_5_-^15^N from Sigma-Aldrich, MO, USA). The values marked with (NMR) were determined separately by proton NMR by the supplier. It is of note that one deuterium at the Cα-position of L-valine-d_8_ was exchanged during syntheses of the standard compounds [3]. | | | |

| **Table S2** Parameters for the mass spectrometric detection of the FTH conjugates resulting from the FITC-mediated cleavage of modified Val residues from Hb and of the respective isotope-labeled standard compounds | | | | | | | | |
| --- | --- | --- | --- | --- | --- | --- | --- | --- |
| **analyte** | **RT** | **transition** | **Q1** | **Q3** | **DP** | **EP** | **CE** | **CXP** |
|  | **min** |  | **m/z** | **m/z** | **V** | **V** | **V** | **V** |
| AA-Val-FTH | 12.34 | AA-quantifier | 560.2 | 445.0 | 190 | 8 | 58 | 20 |
|  |  | AA-qualifier1 |  | 374.0 | 190 | 8 | 61 | 20 |
|  |  | AA-qualifier2 |  | 358.0 | 190 | 8 | 80 | 20 |
| AA-d_7_-Val-FTH | 12.29 | d_7_-AA-quantifier | 567.2 | 445.0 | 190 | 8 | 58 | 20 |
|  |  | d_7_-AA-qualifier1 |  | 374.0 | 190 | 8 | 61 | 20 |
|  |  | d_7_-AA-qualifier2 |  | 358.0 | 190 | 8 | 80 | 20 |
| GA-Val-FTH | 11.70 | GA-quantifier | 576.2 | 531.0 | 200 | 8 | 49 | 20 |
|  |  | GA-qualifier |  | 489.0 | 200 | 8 | 56 | 20 |
| GA-d_7_-Val-FTH | 11.64 | d_7_-GA-quantifier | 583.2 | 538.0 | 200 | 8 | 49 | 20 |
|  |  | d_7_-GA-qualifier1 |  | 489.0 | 200 | 8 | 56 | 20 |
|  |  | d_7_-GA-qualifier2 |  | 496.1 | 200 | 8 | 48 | 20 |
| DHP-Val-FTH | 12.03 | DHP-quantifier | 563.3 | 503.2 | 215 | 10 | 51 | 10 |
|  |  | DHP-qualifier1 |  | 447.0 | 215 | 10 | 56 | 10 |
|  |  | DHP-qualifier2 |  | 489.1 | 215 | 10 | 55 | 10 |
| DHP-d_7_-Val-FTH | 11.98 | d_7_-DHP-quantifier | 570.3 | 503.2 | 215 | 10 | 52 | 10 |
|  |  | d_7_-DHP-qualifier1 |  | 447.0 | 215 | 10 | 56 | 10 |
|  |  | d_7_-DHP-qualifier2 |  | 496.1 | 215 | 10 | 51 | 10 |
| FFA-Val-FTH | 17.96 | FFA-quantifier | 569.1 | 390.0 | 150 | 10 | 70 | 20 |
|  |  | FFA-qualifier1 |  | 81.1 | 150 | 10 | 100 | 10 |
|  |  | FFA-qualifier2 |  | 445.1 | 150 | 10 | 54 | 10 |
| FFA-d_7_-Val-FTH | 17.92 | d_7_-FFA-quantifier | 576.1 | 390.0 | 150 | 10 | 70 | 20 |
|  |  | d_7_-FFA-qualifier1 |  | 81.1 | 150 | 10 | 100 | 10 |
|  |  | d_7_-FFA-qualifier2 |  | 445.1 | 150 | 10 | 54 | 10 |
| IES-Val-FTH | 20.08 | IES-quantifier | 635.1 | 445.0 | 150 | 10 | 57 | 24 |
|  |  | IES-qualifier1 |  | 390.1 | 150 | 10 | 48 | 17 |
|  |  | IES-qualifier2 |  | 359.0 | 150 | 10 | 85 | 23 |
| [^13^C_5_,^15^N]IES-Val-FTH | 20.08 | ^13^C^15^N-IES-quantifier | 641.2 | 448.0 | 150 | 10 | 57 | 24 |
|  |  | ^13^C^15^N-IES-qualifier1 |  | 390.1 | 150 | 10 | 48 | 17 |
|  |  | ^13^C^15^N-IES-qualifier2 |  | 359.0 | 150 | 10 | 85 | 23 |
| Bn-Val-FTH | 19.19 | Bn-quantifier | 579.3 | 444.9 | 270 | 10 | 55 | 25 |
|  |  | Bn-qualifier1 |  | 358.0 | 270 | 10 | 82 | 25 |
|  |  | Bn-qualifier2 |  | 390.0 | 270 | 10 | 70 | 25 |
| [^13^C_5_,^15^N]Bn-Val-FTH | 19.19 | ^13^C^15^N-Bn-quantifier | 585.2 | 448.1 | 240 | 10 | 54 | 25 |
|  |  | ^13^C^15^N-Bn-qualifier1 |  | 359.0 | 240 | 10 | 84 | 25 |
|  |  | ^13^C^15^N-Bn-qualifier2 |  | 390.0 | 240 | 10 | 70 | 25 |
| Ko-Val-FTH | 20.32 | Ko-quantifier | 615.3 | 529.1 | 270 | 10 | 60 | 25 |
|  |  | Ko-qualifier1 |  | 489.1 | 270 | 10 | 53 | 25 |
|  |  | Ko-qualifier2 |  | 374.1 | 270 | 10 | 80 | 25 |
| [^13^C_5_,^15^N]Ko-Val-FTH | 20.32 | ^13^C^15^N-Ko-quantifier | 621.3 | 532.1 | 240 | 5 | 64 | 20 |
|  |  | ^13^C^15^N-Ko-qualifier1 |  | 495.2 | 240 | 5 | 53 | 20 |
|  |  | ^13^C^15^N-Ko-qualifier2 |  | 375.1 | 240 | 5 | 84 | 20 |
| KP-Val-FTH | 16.97 | Kp-quantifier | 573.3 | 489.1 | 240 | 10 | 50 | 25 |
|  |  | Kp-qualifier |  | 374.0 | 240 | 10 | 70 | 25 |
| [^13^C_5_,^15^N]Kp-Val-FTH | 16.97 | ^13^C^15^N-Kp-quantifier | 579.2 | 495.2 | 240 | 10 | 48 | 25 |
|  |  | ^13^C^15^N-Kp-qualifier |  | 375.0 | 240 | 10 | 65 | 25 |

| **Table S3** Peak area ratios for two transitions of the FTH analytes (and their respective isotope-labeled standard compounds) used as quantifiers and qualifiers in the presence of an Hb background after Edman degradation (matrix +) or injected directly (matrix -). | | | | | |
| --- | --- | --- | --- | --- | --- |
| **adduct** | **transition 1 (t_1_)** | **transition 2 (t_2_)** | **matrix** | **ratio (t_1_/t_2_)*^a^*** | **n** |
| AA-Val-FTH | 560.2 → 445.0 | 560.2 → 374.0 | + | 1.32 +/- 0.09 | 14 |
| AA-Val-FTH | 560.2 → 445.0 | 560.2 → 374.0 | - | 1.29 +/- 0.03 | 6 |
| AA-d_7_-Val-FTH | 567.2 → 445.0 | 567.2 → 374.0 | + | 1.15 +/- 0.08 | 13 |
| AA-d_7_-Val-FTH | 567.2 → 445.0 | 567.2 → 374.0 | - | 1.22 +/- 0.04 | 5 |
| GA-Val-FTH | 576.2 → 531.0 | 576.2 → 489.0 | + | 1.87 +/- 0.07 | 10 |
| GA-Val-FTH | 576.2 → 531.0 | 576.2 → 489.0 | - | 1.91 +/- 0.03 | 6 |
| GA-d_7_-Val-FTH*^b^* | 583.2 → 538.0 | 583.2 → 489.0/496.1 | + | 2.09 +/- 0.04 | 10 |
| GA-d_7_-Val-FTH | 583.2 → 538.0 | 583.2 → 489.0/496.1 | - | 2.15 +/- 0.06 | 6 |
| DHP-Val-FTH | 563.3 → 447.0 | 563.3 → 503.2 | + | 2.92 +/- 0.97 | 18 |
| DHP-Val-FTH | 563.3 → 447.0 | 563.3 → 503.2 | - | 2.74 +/- 0.14 | 6 |
| DHP-d_7_-Val-FTH | 570.3 → 447.0 | 570.3 → 503.0 | + | 2.89 +/- 0.12 | 18 |
| DHP-d_7_-Val-FTH | 570.3 → 447.0 | 570.3 → 503.0 | - | 2.67 +/- 0.09 | 5 |
| FFA-Val-FTH | 569.1 → 390.0 | 569.1 → 81.1 | + | 11.8 +/- 1.5 | 16 |
| FFA-Val-FTH | 569.1 → 390.0 | 569.1 → 81.1 | - | 13.0 +/- 0.3 | 6 |
| FFA-d_7_-Val-FTH | 576.1 → 390.0 | 576.1 → 81.1 | + | 12.0 +/- 1.0 | 13 |
| FFA-d_7_-Val-FTH | 576.1 → 390.0 | 576.1 → 81.1 | - | 12.1 +/- 0.3 | 6 |
| IES-Val-FTH*^c^* | 635.1 → 445.0 | 635.1 → 390.0 | + | n.d. |  |
| IES-Val-FTH*^c^* | 635.1 → 445.0 | 635.1 → 390.0 | - | n.d. |  |
| [^13^C_5_,^15^N]IES-Val-FTH | 641.2 → 448.0 | 641.2 → 390.0 | + | 4.77 +/- 0.14 | 18 |
| [^13^C_5_,^15^N]IES-Val-FTH | 641.2 → 448.0 | 641.2 → 390.0 | - | 5.18 +/- 0.09 | 5 |
| Bn-Val-FTH | 579.3 → 444.9 | 579.3 → 358.0 | + | 1.57 +/- 0.20 | 18 |
| Bn-Val-FTH | 579.3 → 444.9 | 579.3 → 358.0 | - | 1.74 +/- 0.03 | 6 |
| [^13^C_5_,^15^N]Bn-Val-FTH | 585.2 → 448.1 | 585.2 → 359.0 | + | 1.66 +/- 0.03 | 18 |
| [^13^C_5_,^15^N]Bn-Val-FTH | 585.2 → 448.1 | 585.2 → 359.0 | - | 1.65 +/- 0.04 | 5 |
| Kp-Val-FTH | 573.3 → 489.1 | 573.3 → 374.0 | + | 1.01 +/- 0.04 | 18 |
| Kp-Val-FTH | 573.3 → 489.1 | 573.3 → 374.0 | - | 1.02 +/- 0.01 | 6 |
| [^13^C_5_,^15^N]Kp-Val-FTH | 579.2 → 495.2 | 579.2 → 375.0 | + | 1.07 +/- 0.03 | 18 |
| [^13^C_5_,^15^N]Kp-Val-FTH | 579.2 → 495.2 | 579.2 → 375.0 | - | 1.10 +/- 0.02 | 5 |
| Ko-Val-FTH | 615.3 → 529.1 | 615.3 → 489.1 | + | 1.16 +/- 0.03 | 18 |
| Ko-Val-FTH | 615.3 → 529.1 | 615.3 → 489.1 | - | 1.17 +/- 0.02 | 6 |
| [^13^C_5_,^15^N]Ko-Val-FTH | 621.3 → 532.1 | 621.3 → 495.2 | + | 1.13 +/- 0.03 | 18 |
| [^13^C_5_,^15^N]Ko-Val-FTH | 621.3 → 532.1 | 621.3 → 495.2 | - | 1.15 +/- 0.03 | 5 |
| *a* The transitions were numbered so that the calculated ratio t_1_/t_2_ was > 1. Usually, the more intense peak in transition 1 was chosen as quantifier signal, except for DHP-Val-FTH. In this case, the peak (*m/z* 563.3 → 503.2) had a higher S/N compared to the peak observed in *m/z* 563.3 → 447.0.  *b* It was reported previously that GA-Val-FTH peaks of the transition *m/z* 576.2 → 489.0 result from two different fragmentations, which can be discerned in the case of the isotope-labeled standard (*m/z* 583.2 → 489.0 and *m/z* 583.2 → 496.1).[4] The sum of the peak areas from the fragmentations (583.2 → 489.0 + 583.2 → 496.1) is approximately equivalent to that of the single peak observed for GA-Val-FTH (*m/z* 576.2 → 489.0). It was used as qualifier signal for the current evaluation, because the Edman matrix interfered strongly with all other fragmentations of GA-Val-FTH.  *c* IES-Val-FTH was not synthesized. | | | | | |

| **Table S4** Levels of the adducts (pmol/g Hb) in erythrocyte samples of human study participants (six non-smokers, 1 – 6, and six smokers, 7 - 12)*^a^* | | | | | | | | | | | | |
| --- | --- | --- | --- | --- | --- | --- | --- | --- | --- | --- | --- | --- |
| **adduct** | **1** | **2** | **3** | **4** | **5** | **6** | **7** | **8** | **9** | **10** | **11** | **12** |
| AA-Val-FTH | 30.6 | 19.3 | 33.0 | 16.4 | 41.9 | 21.1 | 74.3 | 62.1 | 770 | 63.6 | 157 | 22.0 |
| GA-Val-FTH | 17.8 | 11.6 | 12.8 | 10.4 | 18.5 | 9.6 | 43.6 | 44.7 | 378 | 21.5 | 82.8 | 12.0 |
| DHP-Val-FTH | 4.7 | 4.7 | 4.1 | 4.6 | 4.7 | 2.2 | 5.8 | 7.6 | 23.5 | 9.5 | 9.9 | 6.8 |
| FFA-Val-FTH | 8.9 | 9.4 | 13.3 | 10.8 | 14.0 | 12.1 | 15.2 | 9.3 | 11.7 | 7.5 | 15.6 | 9.0 |
| IES-Val-FTH*^b^* | 0.029 | 0.054 | 0.030 | 0.058 | 0.034 | 0.020 | 0.022 | 0.013 | 0.034 | 0.018 | 0.019 | n.d.*^c^* |
| Bn-Val-FTH | 0.24 | 0.20 | 0.06 | 0.37 | 0.11 | 0.25 | 0.11 | 0.14 | 0.08 | 0.05 | 0.12 | 0.06 |
| Ko-Val-FTH | 2.84 | 2.65 | 2.40 | 2.24 | 2.64 | 2.17 | 2.76 | 2.26 | 3.01 | 2.31 | 1.70 | 2.64 |
| Kp-Val-FTH | 4.16 | 4.33 | 4.62 | 3.59 | 4.26 | 3.49 | 4.48 | 3.80 | 4.17 | 4.13 | 5.11 | 3.59 |
| *a* Values are means of duplicates determined on different days.  *b* In each of the duplicate analyses of participants 2, 4 and 5, one signal was above the LOQ and one between the LOD and the LOQ. In four analyses (participants 1, 3, 7 and 9) both signals were between the LOD and the LOQ. In four analyses (participants 6, 8, 10 and 11), one signal each was below the LOD and the other signal between the LOD and the LOQ. After visual inspection, all signals were included for the current evaluation, except those of participant 12.  c not detectable (both signals below the LOD) | | | | | | | | | | | | |

**FFA-Val experiment S1: The effect of different acids used for the solid-phase extraction**

*Main question*

Do the changes of the analytical conditions from the previous method (using cyanoacetic acid for the SPE and a Hypersil GOLD column for the chromatography described by Monien et al. [5]) to the current multimethod (using formic acid for the SPE and an HSS T3 column for the chromatography; Gauch et al.) have an effect on the quantification of FFA-Val?

*Experimental details*

FFA-Val levels were determined fivefold in samples of pooled erythrocytes using **formic acid** according to the method described in the current work. Another five samples of the erythrocyte pool were prepared using 0.25% **cyanoacetic acid** in water/acetonitrile (4:6) for the elution from SPE columns after the Edman degradation as described in Monien et al. [5]. Both sample sets were analyzed with the chromatographic technique employed in the current work by Gauch et al. using an HSS T3 column (1.8 µm, 2.1 mm x 150 mm, Waters).

*Results and interpretation*

Table S5 summarizes the FFA-Val levels. The most important observation was as follows. The mean FFA-Val levels were lower when **formic acid** was used for the SPE compared to the application of **cyanoacetic acid**. The mean peak area ratio 569.1 → 390.1/569.1 → 81.1 was 12.6% lower compared to the respective value determined after direct injection of blank FFA-Val-*d*_7_-FTH (576.1 → 390.1/576.1 → 81.1 = 11.9). This indicated the presence of an overlapping signal in the transition 569.1 → 390.1 leading to a slight overestimation of the FFA-Val levels if cyanoacetic acid was used for the sample workup.

Analyzing the samples using the chromatographic method published by Monien et al. using a Hypersil Gold column (1.9 μm, 2.1 × 150 mm; Thermo Scientific) and a shorter elution profile suggested that there was no effect of the chromatographic changes on resulting FFA-Val levels [5].

| **Table S5** FFA-Val levels in samples of pooled erythrocytes determined using **formic acid** or **cyanoacetic acid** for the SPE after the Edman degradation. Both sample sets were analyzed using the chromatographic techniques described in the current work. | | |  |
| --- | --- | --- | --- |
| **sample name** | FFA-Val  (pmol/g Hb) | peak area ratio  (569.1 → 390.1/569.1 → 81.1) |  |
| **formic acid** |  |  |  |
| Hb_FA_1 | 10.85 | 12.7 |  |
| Hb_FA_2 | 9.72 | 12.9 |  |
| Hb_FA_3 | 10.69 | 13.5 |  |
| Hb_FA_4 | 9.44 | 10.7 |  |
| Hb_FA_5 | 9.16 | 11.7 |  |
| **mean** | **10.0** | **12.3** |  |
|  |  |  |  |
| **cyanoacetic acid** |  |  |  |
| Hb_CAA_1 | 11.9 | 14.7 |  |
| Hb_CAA_2 | 10.2 | 12.4 |  |
| Hb_CAA_3 | 11.0 | 12.4 |  |
| Hb_CAA_4 | 11.6 | 14.2 |  |
| Hb_CAA_5 | 11.3 | 13.2 |  |
| **mean** | **11.2** | **13.4** |  |

**FFA-Val experiment S2: Stability of FFA-Val in hemoglobin**

*Main question*

Is FFA-Val in hemoglobin stable if stored at - 80°C for two years?

*Experimental details*

FFA-Val levels were determined in 12 samples from the Risks and Benefits of a Vegan Diet (RBVD) study as described in **Monien et al.** [5]; and 26 months later the analyses were repeated using the same conditions and, in addition, with the method described in the current work, **Gauch et al.**.

*Results*

The FFA-Val levels are summarized in Table S6. Repeating FFA-Val analyses 26 months after the initial quantification under the same conditions, a mean decrease of FFA-Val levels of about 9% was noted. This suggested that FFA-Val is not entirely stable under the storage conditions. (The erythrocyte samples were homogenized thoroughly before the preparation of the sample aliquots, which were stored at – 80 °C. The repeated determination of the hemoglobin content after 26 months demonstrated the homogeneity of the samples.) It is of note that changing the chromatographic conditions in this re-determination to the HSS T3 column used by Gauch et al. did not change the result significantly (the decrease of FFA-Val levels determined in 2019 and 2021 was 12.4%, data not shown).

The analysis of FFA-Val with the current method (**Gauch et al.**) showed a mean decrease of 21.2% in comparison to the initial measurements in 2019. In addition to the sample ageing, this difference between the two results was probably due to the use of cyanoacetic acid by Monien et al. and formic acid by Gauch et al.. It confirms the observation from experiment S1.

| **Table S6** FFA-Val levels in 12 samples of the RBVD study determined in 2019 using cyanoacetic acid (**Monien et al.**) for the SPE after the Edman degradation. The analysis was repeated in 2021 using the chromatographic technique described in the current work (HSS T3, **Gauch et al.**) and that described in the previous work on FFA-Val (Hypersil GOLD, **Monien et al.**) [5]. | | | | | |
| --- | --- | --- | --- | --- | --- |
| **method** | **Monien et al.** | **Monien et al.** | | **Gauch et al.** | |
| **sample preparation** | January 2019 | March 2021 | | March 2021 | |
| **sample** | FFA-Val | FFA-Val | deviation | FFA-Val | deviation |
|  | pmol/g Hb | pmol/g Hb | % | pmol/g Hb | % |
| **1** | 19.4 | 13.5 | - 30.6 | 12.6 | - 35.0 |
| **2** | 15.4 | 17.8 | + 15.6 | 13.6 | - 11.8 |
| **3** | 20.6 | 20.4 | - 0.7 | 17.4 | - 15.4 |
| **4** | 19.1 | 17.6 | - 8.1 | 17.5 | - 8.6 |
| **5** | 21.1 | 17.3 | - 18.2 | 15.8 | - 25.3 |
| **6** | 15.3 | 11.9 | - 22.5 | 7.9 | - 48.2 |
| **7** | 14.2 | 13.4 | - 5.6 | 13.4 | - 5.4 |
| **8** | 11.1 | 10.9 | - 1.4 | 9.2 | - 17.1 |
| **9** | 14.4 | 13.6 | - 6.0 | 12.2 | - 15.8 |
| **10** | 12.5 | 11.3 | - 9.4 | 9.0 | - 27.5 |
| **11** | 14.9 | 12.7 | - 15.0 | 10.4 | - 30.3 |
| **12** | 12.7 | 12.0 | - 5.2 | 10.9 | - 14.3 |
|  |  |  |  |  |  |
|  |  | **mean** | **- 8.9** |  | - **21.2** |
|  | | | | | |

**References**

1. Bergau N, Herfurth UM, Sachse B, Abraham K, Monien BH. Bioactivation of estragole and anethole leads to common adducts in DNA and hemoglobin. Food Chem Toxicol. 2021;153:112253.

2. Degner A, Carlsson H, Karlsson I, Eriksson J, Pujari SS, Tretyakova NY, et al. Discovery of Novel N-(4-Hydroxybenzyl)valine Hemoglobin Adducts in Human Blood. Chem Res Toxicol. 2018;31:1305-14.

3. Hielscher J, Monien BH, Abraham K, Jessel S, Seidel A, Lampen A. An isotope-dilution UPLC-MS/MS technique for the human biomonitoring of the internal exposure to glycidol via a valine adduct at the N-terminus of hemoglobin. J Chromatogr B. 2017;1059:7-13.

4. von Stedingk H, Rydberg P, Törnqvist M. A new modified Edman procedure for analysis of N-terminal valine adducts in hemoglobin by LC-MS/MS. J Chromatogr B Analyt Technol Biomed Life Sci. 2010;878:2483-90.

5. Monien BH, Bergau N, Hogervorst JGF, Nawrot TS, Trefflich I, Weikert C, et al. Detection of a Hemoglobin Adduct of the Food Contaminant Furfuryl Alcohol in Humans: Levels of N-((Furan-2-yl)methyl)-valine in two Epidemiological Studies. Mol Nutr Food Res. 2021:e2100584.
